# Variation in Lingual Nerve Course: A Human Cadaveric Study

**DOI:** 10.1371/journal.pone.0162773

**Published:** 2016-09-23

**Authors:** Samah M. Al-Amery, Phrabhakaran Nambiar, Murali Naidu, Wei Cheong Ngeow

**Affiliations:** 1 Dept. of Oro-Maxillofacial Surgical and Medical Sciences, Faculty of Dentistry, University of Malaya, 50603, Kuala Lumpur, Malaysia; 2 Dept. of Anatomy, Faculty of Medicine, University of Malaya, 50603, Kuala Lumpur, Malaysia; University of Palermo, ITALY

## Abstract

The lingual nerve is a terminal branch of the mandibular nerve. It is varied in its course and in its relationship to the mandibular alveolar crest, submandibular duct and also the related muscles in the floor of the mouth. This study aims to understand the course of the lingual nerve from the molar area until its insertion into the tongue muscle. This cadaveric research involved the study of 14 hemi-mandibles and consisted of two parts: (i) obtaining morphometrical measurements of the lingual nerve to three landmarks on the alveolar ridge, and (b) understanding non-metrical or morphological appearance of its terminal branches inserting in the ventral surface of the tongue. The mean distance between the fourteen lingual nerves and the alveolar ridge was 12.36 mm, and they were located 12.03 mm from the lower border of the mandible. These distances were varied when near the first molar (M1), second molar (M2) and third molar (M3). The lingual nerve coursed on the floor of the mouth for approximately 25.43 mm before it deviated toward the tongue anywhere between the mesial of M1 and distal of M2. Thirteen lingual nerves were found to loop around the submandibular duct for an average distance of 6.92 mm (95% CI: 5.24 to 8.60 mm). Their looping occurred anywhere between the M2 and M3. In 76.9% of the cases the loop started around the M3 region and the majority (69.2%) of these looping ended at between the first and second molars and at the lingual developmental groove of the second molar. It gave out as many as 4 branches at its terminal end at the ventral surface of the tongue, with the presence of 2 branches being the most common pattern. An awareness of the variations of the lingual nerve is important to prevent any untoward complications or nerve injury and it is hoped that these findings will be useful for planning of surgical procedures related to the alveolar crest, submandibular gland/ duct and surrounding areas.

## Introduction

The lingual nerve (LN) is one of the two terminal branches of the posterior division of the mandibular nerve. It supplies the general sensation to the mucosa of the anterior two-thirds of the tongue, the sublingual mucosa, the mandibular lingual gingiva and the floor of the mouth [1–4].The chorda tympani nerve, a branch of the facial nerve joins this nerve carrying taste fibers from the anterior two third of the tongue and parasympathetic fibers to the submandibular ganglion [4,5]

This nerve occasionally communicates with the inferior alveolar, auriculotemporal or the mylohyoid nerves, and then passes between the lateral and medial pterygoid muscles in the infratemporal fossa before proceeding anteriorly and inferiorly on the surface of the medial pterygoid muscle. It has been found to have variable relationships with the medial surface of the medial pterygoid muscle during its course [6,7]. The LN courses closer to the medial surface of the mandibular ramus until it is just a few millimeters below and behind the junction of the vertical and horizontal rami of the mandible [8]. It enters the submandibular region after passing forward and medially, inferior to the lower border of the superior constrictor muscle of the pharynx and then becomes in close relationship to the lower third molar [1].

It then courses along the periosteum on the medial surface of the mandible to lie opposite the posterior root of the lower third molar. Here, it is covered only by the gingival mucoperiosteum, which is closely bound to the lingual plate of the mandible for a distance of 28.17 mm [6,9,10]. It has been reported that in 20–62% of the time, the LN is in contact with the lingual cortical plate [10–14]. Furthermore, in between 4.6% and 21.0% of the cases, the LN may be situated at/or above the crest of bone [10,11,13–16]. Interestingly the less common site is at the retromolar pad region, which was noticed in 0.15 and 1.5% of the cases [11,13]. When not in contact with the lingual plate, the LN usually lies within 2.28–16.8 mm below the alveolar crest and 0.57–7.10 mm medial from the lingual plate [6,11,17]. Trost *et al*. found that the LN can be very close to the periosteum with a mean horizontal distance of only 1.9 mm but did not report the vertical relationship [18]. The average shortest distance between the LN and the retromolar region was 4.45–8.62 mm [6,7,14–16,19]. The average distance from the mesial and the distal portion of the mandibular third molar area to the LN has been reported to be 9.5 mm and 15.5 mm, respectively [19]. The variations in mean distances of lingual nerve to the mandible as reported by several researchers are summarized in Table 1.

**Table 1:** Mean horizontal and vertical distances of lingual nerve from the third molar bone crest.

Researchers/ Number of nerve	Measurements	
	Horizontal distance	Vertical distance
Behnia *et al*. (2000)	2.06 ± 1.10 mm	3.01 ± 0.42 mm
(n = 669)	(0.00 to 3.20 mm)	(1.70 to 4.00 mm)
Bokindo *et al*. (2015)	7.10 ± 2.80 mm	10.30 ± 5.20 mm
(n = 30)	(1.30 to 15.60 mm)	(2.80 to 19.90 mm)
Diaz *et al*. (2015)	0.57 ± 0.56 mm	9.15 ± 3.87 mm
(n = 46)	(0.00 to 2.50 mm)	(2.33 to 17.50 mm)
Erdogmus *et al*. (2008)	9.30 ± 2.10 mm	7.06 ± 1.30 mm
(n = 42)	(5.20 to 16.20 mm)	(5.01 to 8.97 mm)
Hölzle *et al*. (2001)	0.86 ± 1.00 mm	7.83 ± 1.65 mm
(n = 86)	(0.00 to 4.00 mm)	(4.50 to 14.00 mm)
Karakas *et al*. (2007)	4.19 ± 1.99 mm	9.50 ± 5.20 mm
(n = 34 cadaveric study)	(1.81 to 8.67 mm)	(1.13 to 17.04 mm)
(n = 256 radiographic study)		
Kiesselbach & Chamberlain (1984)	0.59 ± 0.90 mm	2.28 ± 1.96 mm
(n = 21)	(0.00 to 3.00 mm)	(2mm above to 7mm below)
Mendes *et al*. (2014)	4.40 ± 2.40 mm	16.8 ± 5.70 mm
(n = 24)	(2.00 to 11.00 mm)	(12.0 to 29.00 mm)
Miloro *et al*. (1997)	2.53 ± 0.67 mm	2.75 ± 0.97 mm
(n = 20)	(0.00 to 4.35 mm)	(1.52 to 4.61 mm)
Pogrel *et al*. (1995)	3.45 ± 1.48 mm	8.32 ± 4.05 mm
(n = 40)	(1.00 to 7.00 mm)	(Range not reported)
Present study (2016)	(distance not	12.36 ± 3.37mm
(n = 14)	measured)	CI: 11.12 to 13.59

The LN may give off a branch to the lingual gingival tissue, extending horizontally from the medial mandibular cortex at the level of the retromolar pad to mesial of the lower first molars-second premolars to supply the lingual periosteum, gingiva, and mucosa that are overlying the medial alveolar process [7,17,19,20]. Kim *et al*. called it “collateral nerve twigs” while Kocabiyik *et al*. proposed that it should be named “the gingival branch of the lingual nerve” [19,20].

From the third molar region, the LN leaves the lingual plate and courses towards the tongue. The mean sagittal distance of the nerve from retromolar area up to its medial bend toward the tongue has been reported to be 21.47 mm [14]. Chan *et al*. reported that 75% of LNs turned toward the tongue at the first and second molar region. The vertical distance from the LN to the mid-lingual cemento-enamel junctions of mandibular molars and premolars has been reported to be 9.6 mm, 13 mm, and 14.8 mm at the second molar, first molar, and second premolar, respectively [21]. The LN then runs anteriorly and medially on the upper surface of the mylohyoid muscle to travel on the external surface of the hyoglossus muscle deep to the submandibular gland. At the anterior border of the hyoglossus the LN turns around the outer side of the submandibular duct (Wharton’s duct) to ascend into the body of the tongue medial to the duct [22].

The LN usually passes lateral to the submandibular duct, winds below it and then passes upwards and forwards on its medial side on its way to the surface of hyoglossus [7,14]. This relationship has been reported to be the norm in 62.5% side cadaver heads, with the remaining 37.5% cases showing the LN crossing above the duct [6]. No constant position has been found to relate the point of intersection as it can happen anywhere from the distal part of the second premolar tooth to the retromolar trigone. One study reported that this intersection often happens around the premolar region [6] while another reported that 55% of the intersection occurred at the level of the third molar tooth or behind it [23]. The length of this intersection has never been reported. The distance between the alveolar plate, at the level of the third molar region, and the sublingual region of intersection was reported to be 22.6 mm [6]. Occasionally, the submandibular duct runs deep in the floor of the mouth, with no relationship with the LN. This accounted for 11.8% of samples in one cadaveric study [14].

The LN may give out branches that communicate with the mylohyoid nerve, sometimes termed as the “mylohyoid or sublingual curl” [19,24,25]. The prevalence of such communication has been reported to range between 12.5% and 33.3% [19,24]. The average length of this communicating nerve ranged from 14.2 mm to 20.1 mm, with an average of 17.2 mm [19,25–27]. In addition, the LN may also communicate with the hypoglossal nerve on the anterior border of the hyoglossus muscle [9,22,28–30]. The prevalence of such communication has been reported to be 40% in a cadaveric study [31].

The submandibular duct on the other hand, emerges from the medial side of the deep lobe of the submandibular gland, inferior to the mylohyoid muscle in the oral cavity and opens in a small sublingual papilla beside the base of the frenulum of the tongue [32,33].

Lingual nerve neuropathy can results from the presence of pathology or following surgical procedures performed to structures adjacent to the vicinity of the LN. As a consequence, patients may suffer from neurosensory disturbance or impaired taste sensation [34–36]. Due to its course and location, the medial aspect of the mandible adjacent to the third molar and the lateral edge of the tongue base are the areas where the LN is most susceptible to surgical or procedural trauma [8,37] The cause for LN injury in the former site is third molar surgery [8,36,38–46], orthognathic surgery [47–50], and occasionally periodontal [51,52] or pre-prosthetic surgery [38]. LN injury at the lateral edge of the tongue base has occasionally been reported to occur in association with the provision of general anaesthesia via submental endotracheal intubation [53] or following trauma that results from endolaryngeal microsurgery (suspended laryngoscopy) [54,55]. As the LN intersects with the submandibular duct in the floor of the mouth, there is a risk of lingual nerve injury during surgical interventions in this region, such as ductoplasty and removal of sialoliths. Likewise, surgical procedures to the lingual nerve in this region will increase the possibility of submandibular duct injury, which may then cause a ranula to develop [56,57].

As shown by the review above, there is a wide variation in the course of the LN at the third molar region up to its intersection with the submandibular duct. Therefore, appreciating variations in the course of the LN is important for achieving successful outcomes in various dental, oncological and reconstructive procedures by minimizing the risk of injuring this nerve. The present cadaveric study aimed to describe and relate in detail the course of the LN in relation to its adjacent structures, beginning at the molar region in relation to the third molar and the alveolar ridge and lower border of the mandible, along the floor of the mouth, and when intersecting with the submandibular duct. Tracking of the terminal branches of LN in the ventral surface of the tongue was also performed.

## Material and Methods

This study received the relevant Institutional Board of Study approval from Faculty of Medicine [968.35] and Faculty of Dentistry [DF DP 1305/0023(P)], University of Malaya. For the human cadavers, the consent was obtained following a protocol set by the University of Malaya when the bodies were donated for teaching/research purposes. The participants/next of kin provided their written or verbal informed consent to using their body for medical research after death.

### Methods

Seven human cadavers (all elderly males) which were stored in 10% formalin were obtained from the Department of Anatomy, Faculty of Medicine, University of Malaya. The mandibles were checked to be free of lesions and had not undergone any surgery or reconstructive procedure at the area of investigation. The presence or absence of the mandibular molar teeth was recorded. In all, these cadavers had altogether 30 missing molars (average ~ 4 molars per cadaver). Therefore in total we had fourteen lingual nerves for our study.

This study consisted of two parts: (i) obtaining morphometrical measurements of the lingual nerve, and (b) obtaining non-metrical or morphological appearance of its terminal branches. The exploration of the LN was achieved through an incision made in the retromolar region, extending until the point where it adopted a horizontal pathway away from the lingual plate, towards the point where it crosses the submandibular duct.

**Fig 1:**
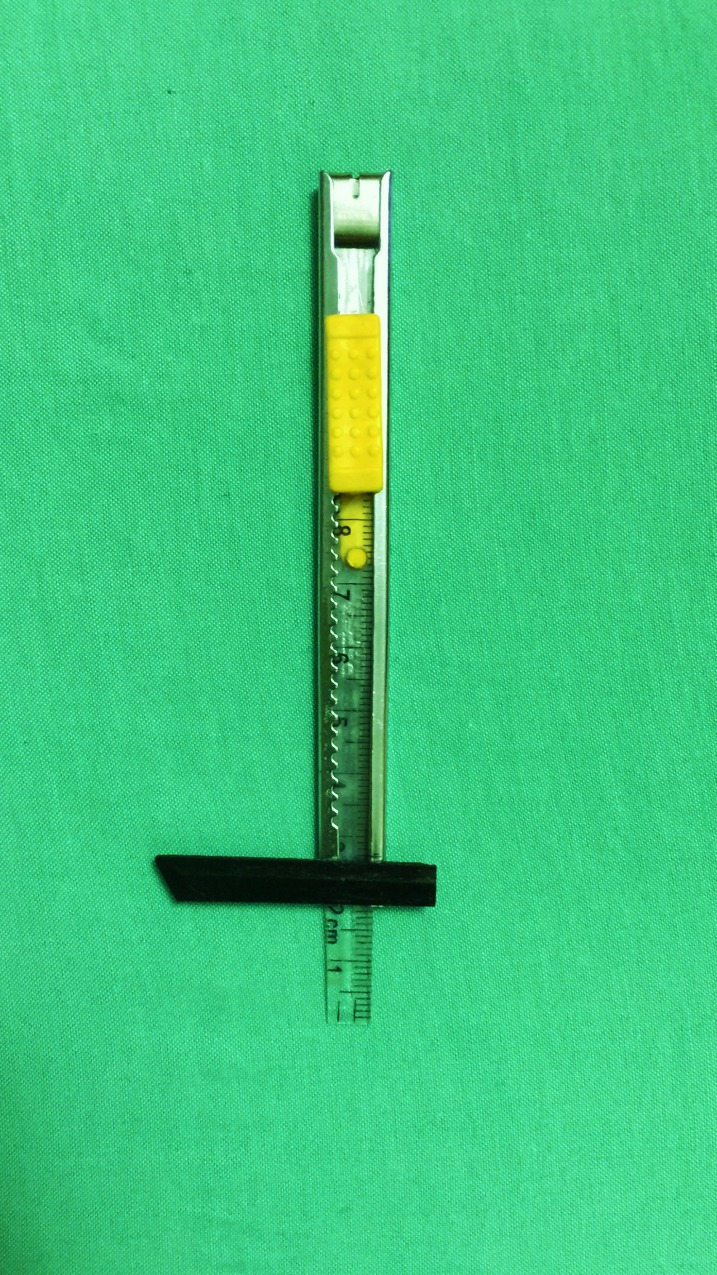
Image showing the custom-made ruler fabricated for this study.

As there was difficulty to insert a standard pair of calipers into the narrow exposed retromolar region and in order to avoid over retracting the soft tissue (which may change the anatomical relation of the lingual nerve), a customized ruler was fabricated to ensure good access and standardization in measuring all the anatomical sites in this study (Fig 1).

The vertical distances from the lingual nerve to two anatomical landmarks, namely the alveolar ridge and the lower border of the mandible, was measured using this measuring device. The dentition on the mandible was used as a position landmark because all mandibles came in different sizes, and it was difficult to standardize the sites for measurement. In cases where posterior teeth were missing, reconstruction of the lost teeth was done by placing average sizes of the molar teeth onto the alveolar ridge. The width or length of each molar was determined using Woelfel's dental anatomy guideline [58] and this position was marked on the alveolar ridge using a permanent marker. The approximate positions of the occlusal surface landmarks (cusps and developmental grooves) were marked accordingly.

#### (a) The distance of lingual nerve to alveolar ridge

**Fig 2:**
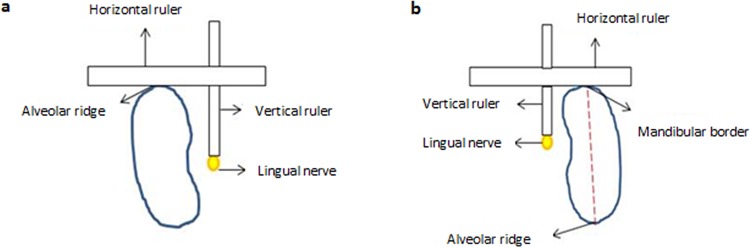
Illustration showing the ruler in use for measuring the distance of the lingual nerve to (a) the alveolar ridge and (b) the lower border of the mandible.

After the three sites of each molar tooth was determined and marked on the alveolar ridge, the horizontal bar of the ruler was placed onto the alveolar ridge and the vertical ruler, which was attached to the horizontal bar of the device, was slid by using the adjacent part until it contacted the lingual nerve. This distance was recorded from the alveolar ridge (Fig 2).

#### (b) The distance of lingual nerve to the inferior mandibular border

The process of obtaining this measurement was made following exposure of the inferior border of the mandible. This measurement was determined as the distance between the lingual nerve and the inferior border of mandibular border (IBM) at the previously determined three points of reference sites marked on the alveolar ridge (Fig 2).

#### (c) The length of lingual nerve prior to diversion to the tongue

**Fig 3:**
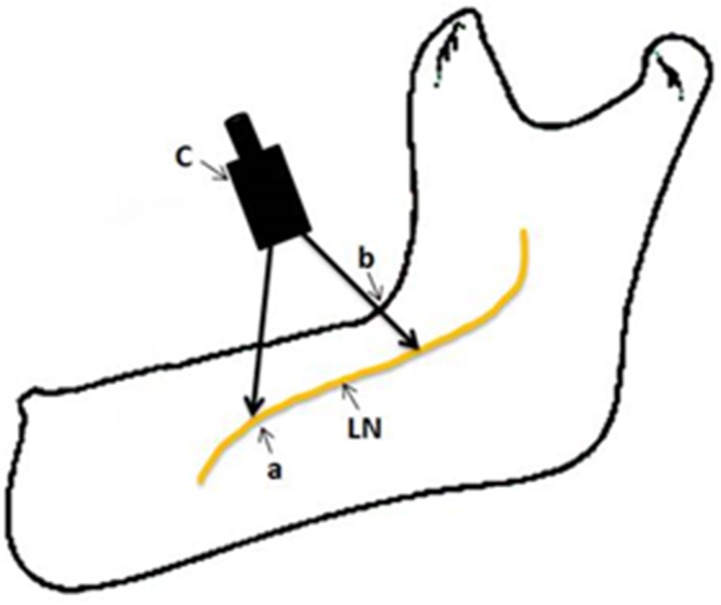
The length of the lingual nerve at the floor of the mouth; a) the point at which the lingual nerve changes direction towards the tongue, b) the deepest point at the angle of the mandible, c) the caliper used to measure the length of lingual nerve.

One other measurement performed was the length of the lingual nerve at the floor of the mouth prior to diversion towards the tongue. This extension of the lingual nerve was measured as a distance from the deepest point at the angle of the ramus and body of the mandible to the point at which it changes its direction toward the tongue (Fig 3).

As the nerve extends anteriorly, it bends and changes its direction to reach the tongue. The location at which the nerve changes its direction varies. These locations were observed in all the 14 cases, and their exact sites were recorded.

The deep incision described above was extended from the point at which the lingual nerve changes its direction toward the tongue. Then, the soft tissue was dissected carefully until the submandibular duct was found, while maintaining its anatomical relationship with the lingual nerve. The presence of the lingual nerve looping around the submandibular duct was first determined.

#### (d) Determining the beginning and the end of overlap between the lingual nerve and submandibular duct

**Fig 4:**
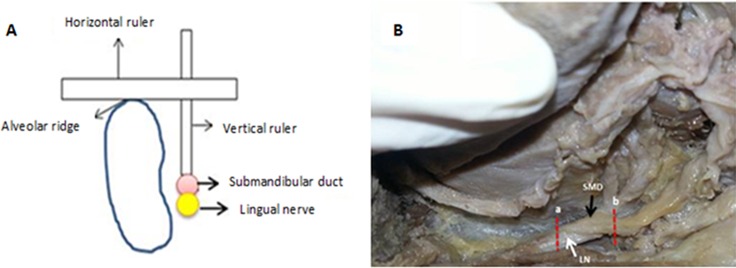
(A) The diagram illustrates the way to determine the start and the end point of overlap between the submandibular duct and the lingual nerve. (B) The start of the overlap (a); the end of overlap (b); LN: lingual nerve; SMD: submandibular duct.

When present, the custom-made vertical ruler was slid down until it touched the starting point of the overlap of the lingual nerve and the submandibular duct. The horizontal ruler was supported on the alveolar ridge as in Method (a). The position at which the horizontal bar rested on the alveolar ridge in relation to the dentition was recorded (Fig 4). These positions were reported as being any of the followings:

1. At the lingual developmental groove of first molar
2. Between first & second molars
3. At the lingual developmental groove of second molar
4. Between second & third molars
5. At the lingual developmental groove of third molar
6. At the distal surface of third molar

The same steps were followed to determine dental relationship at the end of this overlap.

#### (e) Measuring the distance of overlap between the lingual nerve and the submandibular duct

**Fig 5:**
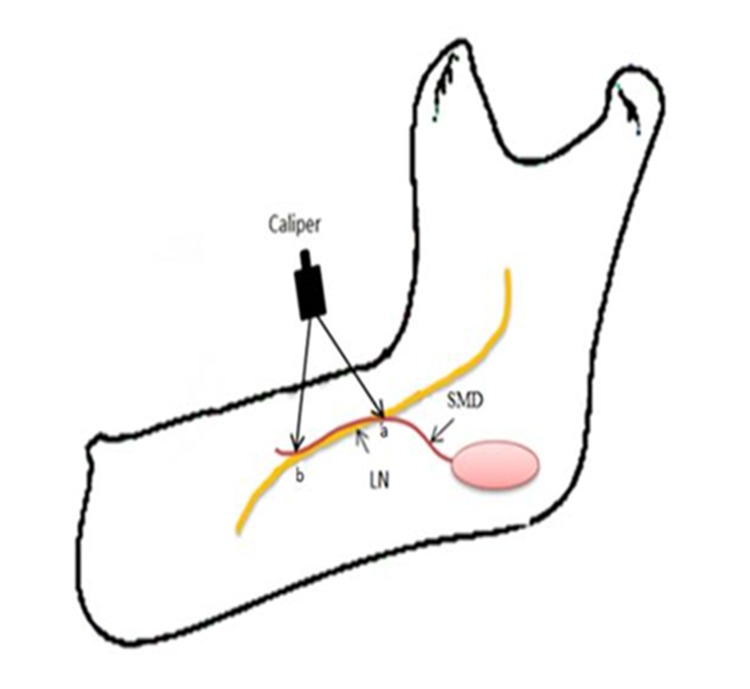
The diagram illustrates the measuring of the extent of overlap between the lingual nerve and the submandibular duct (distance a-b).

Using the landmarks identified in Method (a), the extent of overlap between the lingual nerve and the submandibular duct was measured from the start to the end of the overlap between the two structures using a pair of manual calipers (Fig 5).

### Non-metrical or morphological considerations prior to insertion into the tongue

**Fig 6:**
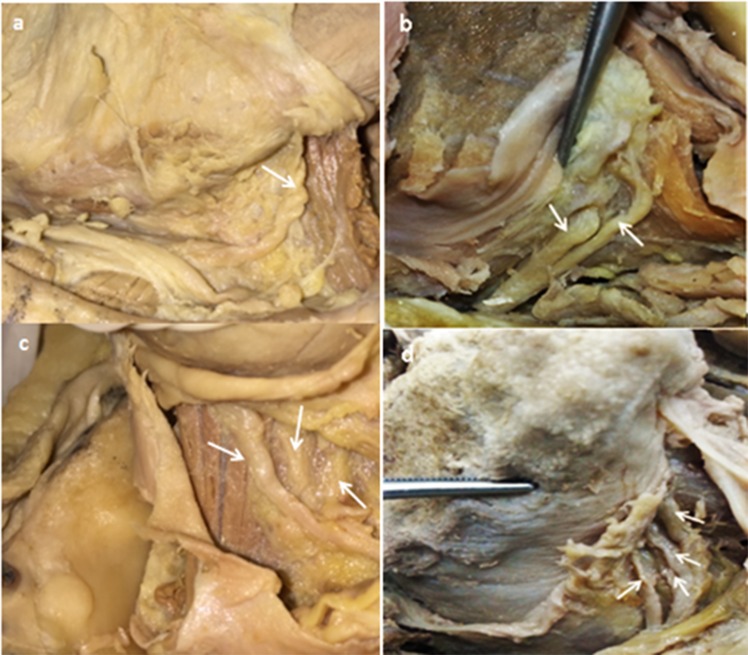
The four different patterns of the lingual nerve endings at the ventral surface of the tongue (a) Only one lingual nerve at the terminal end; (b) Two branches were found at the terminal end; (c) Three branches were found at the terminal end; (d) Four branches or more were found at the terminal end.

As the nerve extends anteriorly, it inserts into the tongue. The location at which the nerve changes its direction to insert into the tongue varies, and all their exact sites were recorded. Furthermore, a detailed study was done on the pattern of the lingual nerve endings at the ventral surface of the tongue (Fig 6). This pattern is described as the followings:

Pattern 1: Only one lingual nerve at the terminal end
Pattern 2: Two branches were found at the terminal end
Pattern 3: Three branches were found at the terminal end
Pattern 4: Four branches or more were found at the terminal end.

## Statistical Analysis

All the measurements were performed by the same examiner (first author) and descriptive analyses reporting on the mean and standard deviations was performed using SPSS 20 program.

## Results

In the present study the mean distance between the LN and the alveolar ridge was 12.36 mm (SD 3.37)[Table 2]. This distance varies between sites, measuring at the third molar region at an average of 12.61 (SD 3.40) mm. At the second molar region, this distance became shorter at 11.46 (SD 2.98) mm before increasing again at the first molar area to 14.38 (SD 4.35) mm.

**Table 2:** The mean distance between the lingual nerve and the 2 anatomical landmarks.

Site	Distance in mm (mean + SD; 95% CI)	
	Alveolar ridge	Lower border of mandible
First molar	14.38 (4.35)	15.00 (2.16)
	CI: 7.46 to 21.29	CI: 11.56 to 18.44
Second molar	11.46 (2.98)	12.46 (3.97)
	CI: 9.66 to 13.26	CI: 10.06 to 14.86
Third molar	12.61 (3.40)	10.79 (3.49)
	CI: 10.64 to 14.57	CI: 8.77 to 12.80
Overall	12.36 (3.37)	12.03 (3.75)
	CI: 11.12 to 13.59	CI: 10.66 to 13.41

In comparison, the distance between the lingual nerve and the inferior mandibular border increased gradually from the third molar region to the first molar region. At the third molar region, this distance was 10.79 (SD 3.49) mm, 12.46 (SD 3.97) mm at the second molar, and 15.00 (SD 2.16) mm at the first molar region. In general, the LN was located 12.03 mm from the lower border of the mandible. Table 2 provides the mean distance as well as 95% confidence interval of all the measurements obtained.

Table 3 outlined the detailed measurements obtained from each cadaver, together with the extent of the lingual nerve on the floor of the mouth prior to diversion towards the tongue. It also provides the number of terminal branches of the lingual nerve.

**Table 3:** The detailed morphometric measurements of each cadaver as obtained when tracing of the course of the lingual nerve. The number of terminal branches is shown at the far right of the table.

	Distance between LN & alveolar ridge (mm)*		Distance between LN & IBM (mm)^φ^		Length of LN on the floor of mouth (mm)		No. of Terminal branches	
No. 1	right	left	right	left	right	left	right	left
**M3**	17	17.5	12	16	41	38	2	2
**M2**	17.5	16	17	15				
**M1**	19	16.5	16	17				
**No. 2**								
**M3**	11.5	14	7	10	23	28	2	3
**M2**	11.5	13	9	13				
**M1**	-	-	-	-				
**No. 3**								
**M3**	10	13	12	14	31	24	3	4
**M2**	12	13	10	16				
**M1**	13	-	12					
**No. 4**				-				
**M3**	15	8	8	12	20	17	2	1
**M2**	8	8	10	13				
**M1**	-	-	-	-				
**No. 5**								
**M3**	15	10	15	10	22	22	2	3
**M2**	12	8	15	13				
**M1**	-	9	-	15				
**No. 6**								
**M3**	7	7	6	4	17	19	4	3
**M2**	9	-	2	-				
**M1**	-	-	-	-				
**No. 7**								
**M3**	15	14.5	12	13	25	29	2	2
**M2**	10	11	15	14				
**M1**	**-**	-	-	-				

IBM: inferior border of mandible; LN: lingual nerve

*No significant difference noticeable between sides (P>0.05)

φ No significant difference noticeable between sides (P>0.05)

**Fig 7:**
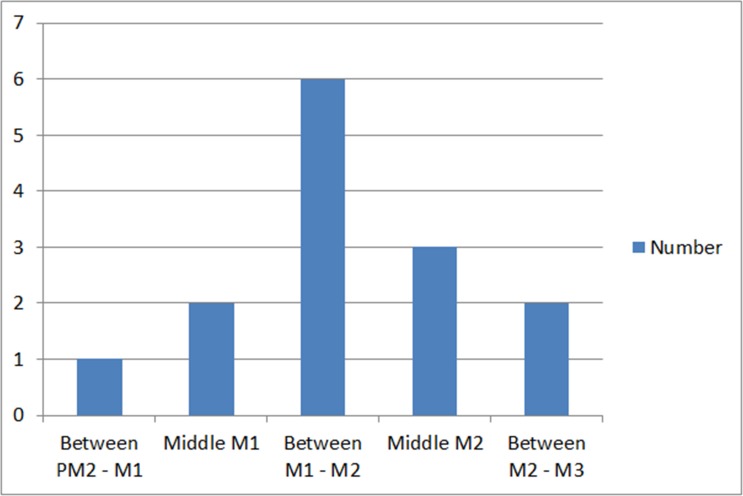
The location at which the lingual nerve made a sudden deviation toward the floor of the mouth.

We are mindful that the LN passes besides the lingual plate in the oral cavity, then lies of the floor of the mouth before it changes direction and deviates toward the tongue. The point at which the lingual nerve changed direction (deviated) varied from one cadaver to another. The mean distance of the LN lying on the floor of the mouth before it curved and deviated toward the tongue was 25.43 (SD 7.31) mm (Table 3). This distance ranged from 17 mm to 41 mm. The 95% confidence interval was 21.21 to 29.65 mm. The five different locations where deviation occurred are summarized in Fig 7.

In all cases, the LN was found to make a sudden deviation towards the floor of the mouth between the mesial of the first molar (M1) and distal of the second molar (M2) teeth regions (Fig 7). In most cases (42.9%), the deviation was at between the proximal surfaces of the first and second molar (M1-M2). The most mesial deviation was noticed a single case, occurring at the mesial aspect of the first molar.

Thirteen lingual nerves were found to overlap or loop around the submandibular duct, i.e. they coursed from the superior to the beneath the submandibular duct. In one case the submandibular duct was surprisingly not traceable. These looping began somewhere between the second and third molars (Table 4). It is evident that the majority of them occurred between the lingual developmental grooves of the second and third molars (92.4%). In10 out of the 13 cases, the loop started around the third molar region but only in one case this loop began at the distal surface of the third molar.

**Table 4:** The location where the looping of the lingual nerve over the submandibular duct began.

Location	Number (percent)
At the lingual developmental groove of second molar	3 (23.1%)
Between second & third molars	4 (30.8%)
At the lingual developmental groove of third molar	5 (38.5%)
At the distal surface of third molar	1 (7.6%)
Total	13 (100%)

The looping of the LN over the submandibular ended somewhere between the first and the third molars region. The majority (69.2%) of these looping ended at between the M1-M2 and at the lingual developmental groove of the second molar. In the remaining 4 cases, 2 of the overlaps terminated at the level of the lingual developmental groove of the first molar, while the remaining two ended this relationship between the second and third molar (Table 5).

**Table 5:** The location where the looping of the lingual nerve over the submandibular duct ended.

Location	Number (percent)
At the lingual developmental groove of first molar	2 (15.4%)
Between first & second molars	5 (38.5%)
At the lingual developmental groove of second molar	4 (30.8%)
Between second & third molar	2 (15.4%)
Total	13 (100%)

In summary, it can be observed that in 76.9% of the cases the loop started around the M3 region but all looping ended at around the first and second molar teeth region, with none of them occurring beyond the lingual groove of M1.

The exact distance of the overlap was measured for each of the 13 cases and a mean distance of 6.92 (SD 2.78) mm, was found. This distance measured as little as 1.50 mm to as much as 13.00 mm.

**Fig 8:**
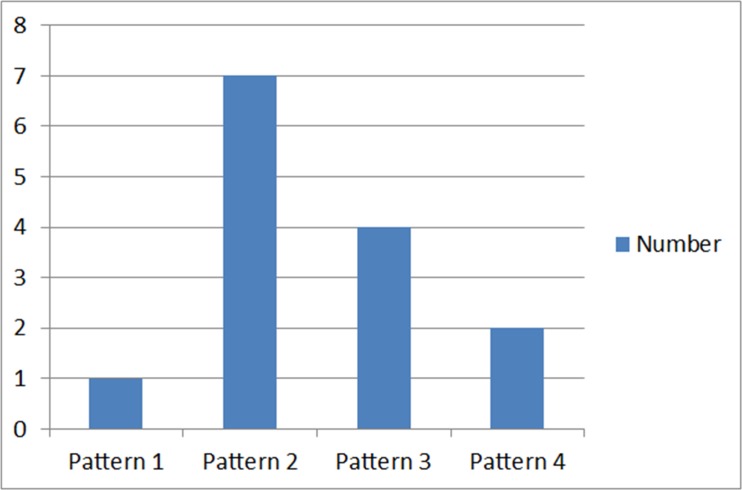
The pattern of terminal branching of the lingual nerve.

At the ventral surface of the tongue, the lingual nerves gave out more than one terminal branch in 13 hemi-mandibles. This study recorded 4 different patterns of nerve endings, as shown in Fig 8.

The most common pattern was the presence of 2 terminal branches at the ventral surface of the tongue, accounting for half of the pattern of branching. The presence of 3 and 4 terminal branches were also evident (28.6% and 14.3% respectively). The least common was the presence of a single terminal branch in one case. Interestingly the findings showed variations within each cadaver (between the right and left side), with only two cadavers showing similar pattern of terminal branching at both sides (Table 3).

## Discussion

The most commonly reported neuropathy of the lingual nerve is associated with surgical trauma that results from removal of the impacted lower third molars [59,60], followed by needle-related injury associated with the provision of inferior alveolar nerve blocks [61–63]. Other less common causes include periodontal and preprosthetic surgery that is performed at the vicinity of the lingual nerve at the retromolar region [38], resection of tumor at the retromolar trigone [64], neural invasion of cancer [65] and orthognathic surgery [47]. Most of these cases of injury or tumor invasion have been reported to happen at the medial aspect of the mandible adjacent to the third molar and the lateral edge of the tongue base [8,37]. One other site where lingual nerve injury has been reported is at the floor of the mouth, as a result of submental endotracheal intubation [53].

As the main cause of LN injury is third molar surgeries, priority of earlier studies was to determine the relationship of the LN to the third molar tooth, as shown in Table 1. In this study, the average vertical distance was greater than the distance recorded by all other authors, apart from Mendes et al. [6] (Table 1). We did not observe any difference between the measurements obtained from the right and left sides of the hemi-mandibles, so all results shown were those of pooled data. We did not measure the distance of the lingual nerve to the lingual bone plate as we found that dissecting cadavers fixed in formalin to find this nerve for vertical measurements will cause distortion to the horizontal relationship. The relationship of this nerve to the other posterior teeth was not given prominence as there is just one study that reported the vertical distance of the LN adjacent to the first, second and third molars [21]. However, this dental relationship may be important clinically as incidences of lingual nerve injury have been reported in relation to vestibuloplasty [66] and implant surgery performed anterior to the third molars [67]. The current study tries to address this shortcoming and provide additional information on Asian subjects, a group of subjects that has not been examined by Chan *et al*. [21].

Chan et al. [21] determined the vertical relationship between the LN and the adjacent and found that it extended to as far anteriorly as the premolar region, before diverting towards the tongue. They noticed that there was an increase in the distance between the LN and the adjacent dentition the more anterior this nerve travelled. They reported that at the second molar, the vertical distance from the cement-enamel junction was 9.6 (SD 3.4) mm and this increased gradually to become 12.95 (SD 4.0) mm at the first molar region and 15 (SD 2.55) mm at the second premolar region; at the left side the nerve extends up to the first premolar region and a distance of 13.2 mm had been recorded. Their measurements differ from those recorded in this study, where the mean distances were 11.65 (SD 3.2) mm and 14.4 (SD 4.3) mm at the second and the first molar region respectively. No lingual nerve was found to be closely related to the premolars in our study. This variation in findings may arise as a result in the selections of the sample, namely racial origin of the cadaver studied. Do note that the LN was closer to the alveolar ridge around the second molar region (at 11.64 mm) as opposed to the first (14.37 mm) or third molars (12.25 mm). This relationship may occur as a result of the presence of the submandibular gland beneath the floor of the mouth that may have displaced the nerve along with the floor, superiorly.

Unlike other studies, the current research also studied the distance of the LN to the inferior border of the mandible. The LN was located progressively away from the inferior border of the mandible beginning from the third molar anteriorly. It was located at 10.5 (SD 3.71) mm from the mandibular inferior border at the third molar, at 12.0 (SD 4.2) mm at the second molar and 15.0 (SD 2.16) mm at the first molar. The LN then runs anteriorly and medially on the upper surface of the mylohyoid muscle, crosses the styloglossus to travel on the external surface of the hyoglossus muscle deep to the submandibular gland. The distance travelled by the nerve before reaching the point at which it deviated sharply toward the tongue is 25.16 (SD 7.8) mm with a range of 17–41 mm. This distance is only slightly higher than the 21.47 mm reported by Hölzle *et al*.[14]. This information shall become useful for any surgeon who explores the submandibular region from an extra oral approach. In addition, this information is important for cosmetic surgeons who plan to shave off the lower border of the mandible to sharpen the shape of the mandible. They will have a mental map of the course of the LN in relationship to the lower border of the mandible, and hence avoid causing any unnecessary iatrogenic injury along its course of 25 mm.

The site where the LN diverts toward the tongue is the site where the submandibular gland is situated. Chan *et al*. (2010) recorded different locations where the LN made a diversion toward the tongue. In their study, most cases showed a sudden change in the direction of the LN at the level of the first molar on both sides of the jaw. In this study, we tried to be more precise when identifying the turning point by describing in detail the site of the molar teeth concerned, namely the mesial-distal contact points and the developmental groove of the relevant teeth. Chan et al. reported that the LN deviated toward the tongue anywhere between distal surface of the second premolar and the mesial surface of the third molar [21]. In our research it was noticed that the proximal area between the first and second molars is the most common site of deviation. It is believed that such difference occurred due to difference in the size of the jaw and floor of the mouth of the cadavers. The cadavers used in this study were Asians, hence they have smaller oral cavity, which may caused the lingual nerve to divert to the tongue earlier (more proximally) than in Caucasians.

Most studies simplified the relationship between the LN and the submandibular duct by describing a loop configuration of the nerve around the duct [68–70]. In more detail, the LN passes lateral to the submandibular duct, winds below it and then passes upwards and forwards on its medial side on its way to the surface of hyoglossus [7,14]. However, Mendes *et al*. reported that this norm only occurred in 62.5% side of cadaver heads. In the remaining 37.5% of cases the LN crossed above the duct [6]. In 12 out of the 13 of the cases in this study, the lingual nerve passed below the submandibular duct before rising again toward the ventral surface of the tongue; this follows the normal pattern described by Mendes *et al*. Occasionally, the submandibular duct runs deep in the floor of the mouth, with no relationship with the lingual nerve. This accounted for 11.8% of samples in a different cadaveric study [14]. We found one such position out of the 14 sites dissected that presented with this relationship.

Mendes *et al*. reported no constant position was found to relate the point where the LN intersects the submandibular duct although this often happens around the premolar region [6]. In contrast, Klepácek and Skulec [71] observed a constant beginning of this association between the two structures in the vicinity of the upper surface of the posterior portion of the mylohyoid muscle close to the inner surface of the apex of the third lower molar socket. The finding of the current study is similar to their finding. The distance of overlap (loop) between these 2 structures was found to be 6.92 (SD 2.78) mm. In a majority (69.62%) of loops, they ended at between M1-M2, and at the lingual developmental groove of M2. It is reported that the high amount of viscous mucuos secretion of the submandibular gland increases the chance of the gland to be affected by the formation of small sialoliths, thus increasing the need for surgical intervention in this area [72,73]. Such surgical procedures if performed without enough appreciation of this complex relationship will increase the possibility of injury to the lingual nerve as it is intertwined with the submandibular duct.

The LN gives out terminal branches when it reaches the ventral surface of the tongue [22,28]. Two patterns were recorded by Rusu et al. [22] with 50% of the cases reporting presence of a single primary trunk while the rest showed two primary trunks. Although both our research and Rusu *et al*.’s had the same sample size, there was a difference between these two studies in the pattern of insertion of the terminal branches into the ventral surface of the tongue. Four difference patterns were recorded in this study. The presence of two terminal branches was the most common pattern and accounted for 50.0% of the cases. In 28.6% of cases, the LN terminated with 3 terminal branches, and in 14.3%, it had four terminal endings.

This different in the patterns at which the nerve ends at the ventral surface of the tongue may be the result of variation in racial ancestry of the sample studied. The anatomical variations were not only restricted between the different cadavers, but also noted within the same cadaver. Beside variations in the metrical measurement, as shown in Table 3, the numbers of the terminal branches of the lingual nerve on the ventral surface of the tongue were different on either sides of cadaver. This difference in pattern, with more branching in Asians may affect the way we use the tongue as a donor site of regional flap.

Lastly, in between 4.6% and 21.0% of cases, the LN was reported to be situated at/or above the crest of bone [10,11,13–16] or at the retromolar pad region in another 0.15–1.5% of cases [11,13]. So far, these variations had only been observed in Caucasian cadavers [19], and rightly so, none was observed in our cadavers. The LN has been reported to give off a branch to the lingual gingiva extending horizontally from the medial mandibular cortex at the level of the retromolar pad to mesial of the lower first molars-second premolars, giving rise to the so called “collateral nerve twigs” [19] or “the gingival branch of the lingual nerve” [20]. It has also been reported to communicate with either the medial or lateral trunk of the hypoglossal nerve on the anterior border of the hyoglossus muscle [9,22,28–30].Both of these variations were not observed in our study.

Given the facts that all these hemi-mandibles came in different sizes, it was difficult to standardize the sites to obtain reliable measurements. Hence we used the dentition on the mandible as a position landmark as we think this is a practical approach with possible clinical application. However, the other limitation of this study is the fact that some of these cadavers presented with missing mandibular molar teeth (30 altogether in 7 cadavers). We are aware that this may affect the reliability of some measurements, hence as outlined in the Methodology section, we tried to standardize our landmarks by reconstructing the missing teeth by placing average sizes for molar teeth onto the alveolar ridge. The width or length of each molar was determined using Woelfel's dental anatomy guideline [58].

One of other limitations of this study is the fact that this is a descriptive cadaveric study, thus it has limited novelty as compared to a meta-analysis. We were limited by the number of cadavers available, and the findings may not be possibly extrapolated to the general population given our limited number of subjects. We have tried to perform a meta-analysis on all currently available data on the lingual nerve but the big difference in the methodology employed by various different researchers make this exercise impossible. Hence, we tried to employ the practical and clinical significance of the lingual nerve when designing this study, i.e. to provide us with additional information to fill our current gaps on knowledge with regards to the lingual nerve in Asians.

## Conclusion

The course of the lingual nerve at the molar region and its pattern of insertion at the ventral surface of the tongue showed great variations—not only between the different cadavers but also within the different sides of the same cadaver. These anatomical variations are assumed to be the possible causes of complications or reasons for injury after injecting local anesthesia or even following normal third molar extraction. This detailed study on the course of the lingual nerve and its relationship to it neighboring structures (including the submandibular duct) will provide a useful reference for preparation of clinical applications and surgical procedures in the floor of the mouth, particularly in the Asian population.
